# Clinical and healthcare burden of disease associated with cytomegalovirus in allogeneic hematopoietic stem cell transplantation – A retrospective single‐center study

**DOI:** 10.1111/tid.13947

**Published:** 2022-09-27

**Authors:** Juha Ranti, Katariina Perkonoja, Tommi Kauko, Riku Korhonen

**Affiliations:** ^1^ Department of Hematology Turku University Hospital Hospital District of Southwest Finland Turku Finland; ^2^ Auria Clinical Informatics Hospital District of Southwest Finland Turku Finland; ^3^ MSD Finland Oy Espoo Finland

**Keywords:** allogeneic stem cell transplantation, CMV, disease burden, hospitalization, healthcare resource utilization, HLA

## Abstract

**Background:**

CMV infection is a common complication in allogeneic hematopoietic stem cell transplantation (HSCT). We investigated the association of clinically significant CMV (CS‐CMV) infection with clinical outcomes and healthcare resource utilization in allogeneic HSCT patients in Finland.

**Methods:**

This retrospective study included adult patients who received their first allogeneic HSCT between January 1, 2013, and December 31, 2018, at the Turku University Hospital. Data were collected from the hospital data lake. Clinical and healthcare outcomes were investigated at one year and mortality up to three years.

**Results:**

The study included 251 patients. CMV seroprevalence was 69.7%. CS‐CMV infection occurred in 59.0% of the patients, and of those, 14.2% had ≥2 infections. The median time to CS‐CMV infection was 34.5 days (Q_1_–Q_3_, 27.0–45.0). Recipient and donor seropositivity, and lymphoproliferative diseases were associated with higher, and HLA identical sibling donors with lower CS‐CMV infection risk. CS‐CMV infection was not associated with mortality in three years of follow‐up. One hundred thirty‐three (89.8%) and 75 (72.8%) patients with and without CS‐CMV infection, respectively, were readmitted to the hospital. Patients with CS‐CMV infection had more hospital readmissions (incidence rate ratio [IRR] 1.38, 95% confidence interval [CI] 1.10–1.73, *p* = .005) and patients with one CS‐CMV infection (IRR 1.48, 95% CI 1.12–1.94, *p* = .005) or ≥2 infections had longer length of hospital stay (IRR 2.71, 95% CI 1.76‐4.35, *p* < .001).

**Conclusion:**

CMV seroprevalence is relatively high among Finnish allogeneic HSCT patients. CS‐CMV infection was common and associated with a higher readmission rate and longer length of hospital stay.

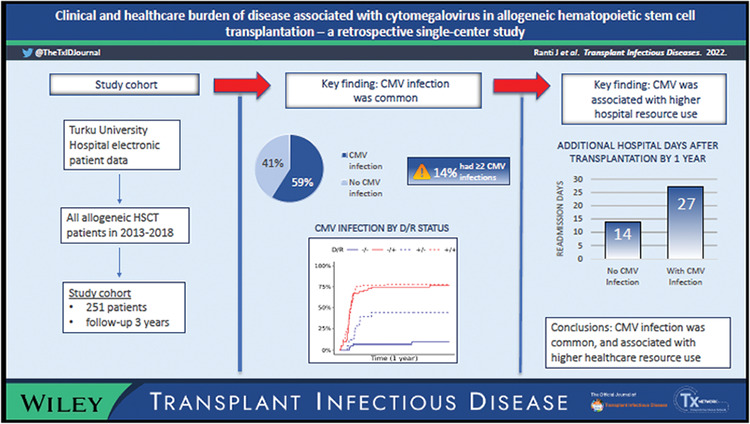

**List of Abbreviations**
CMV: cytomegalovirus
CS: clinically significant
D: donor
GVHD: graft‐versus‐host disease
HR: hazard ratio
HSCT: hematopoietic stem cell transplantation
IRR: incidence rate ratio
OR: odds ratio
PCR: polymerase chain reaction
PET: pre‐emptive therapy
Q_1_–Q_3_
: lower and upper quartiles
R: recipient

## 1 INTRODUCTION

CMV infection is a common complication in allogeneic hematopoietic stem cell transplantation (HSCT).^1^, ^2^ CMV infection occurs in 30%–70% of the patients, usually within 0–100 days after the transplantation,^3^, ^4^, ^5^, ^6^, ^7^ but late CMV infection (after day 100 post allogeneic HSCT) may occur in 10%–30% of the patients.^3^, ^8^ When untreated, CMV infection leads to CMV disease, most commonly pneumonia or gastroenteritis, in 10%–40% of the patients and CMV disease is associated with higher mortality.^2^ Both early and late CMV infection are associated with increased morbidity and non‐relapse mortality.^4^, ^5^, ^9^

Allogeneic HSCT patients are commonly monitored for CMV replication by polymerase chain reaction (PCR) in blood or urine samples. If clinically significant CMV (CS‐CMV) infection is observed, pre‐emptive therapy (PET) with antivirals is often initiated to prevent CMV disease. Established anti‐CMV drugs include ganciclovir^10^ or valganciclovir,^11^, ^12^ foscarnet,^13^ and cidofovir as second‐line treatment.^14^ The introduction of PET has reduced the incidence of CMV disease down to 1%–10%.^4^, ^5^, ^15^, ^16^

Donor (D) and recipient (R) CMV serostatus are significant risk factors for CMV infection in allogeneic HSCT,^2^, ^4^, ^5^ and D/R status is associated with CMV‐related outcomes, such as delayed CMV‐specific immune reconstitution, repeated CMV infections, additional anti‐CMV treatment courses, late CMV infection and development of CMV disease.^3^, ^17^, ^18^, ^19^, ^20^, ^21^ Other risk factors include the use of high‐dose corticosteroids, T‐cell depletion, acute and chronic graft‐versus‐host‐disease (GVHD), and the use of mismatched or unrelated donors.^8^, ^15^, ^21^, ^22^, ^23^, ^24^, ^25^

CMV infection, CMV disease, and PET have been reported to associate with higher clinical burden and increased healthcare resource utilization. Retrospective single‐center studies have reported that a greater economic burden is associated with CMV treatment.^26^, ^27^, ^28^ Three recent studies from the United States reported the association of CMV infection with higher readmission rates and greater healthcare costs, driven by medication costs, additional hospital days, and outpatient visits.^29^, ^30^, ^31^

There are no published data on CMV seroprevalence or CS‐CMV infection among allogeneic HSCT patients in Finland. A study from the Finnish Maternity Cohort serum bank reported a decline in CMV seroprevalence from 84.5% in 1992 to 71.5% in 2012.^32^ Pregnant women are typically younger than allogeneic HSCT patients, and they may not reflect the seroprevalence in allogeneic HSCT patients. Also, we don't have published data on the healthcare resource utilization associated with CMV infection in allogeneic HSCT in Finland. In this retrospective study from secondary patient data, we investigated the association of CS‐CMV infection with clinical outcomes and healthcare resource utilization in the treatment of CMV in allogeneic HSCT patients in Finland.

## 2 MATERIALS AND METHODS

### 2.1 The study population and data collection

This single‐center, retrospective, observational cohort study with Finnish allogeneic HSCT patients investigated the association of CS‐CMV infection with clinical outcomes and healthcare resource utilization. The study included adult patients who received their first allogeneic HSCT [10th revision of the International Statistical Classification of Diseases and Related Health Problems (ICD‐10) code: Z94.8, Nordic Classification of Surgical Procedures 2015 codes: WW202/204/206/210, ≥18 years of age] between January 1, 2013, and December 31, 2018, at the Hospital District of Southwest Finland. Patients who had participated or were considered to participate in the letermovir phase 3 CMV reactivation prophylaxis study^33^ were excluded. Patients were followed for 3 years after the transplantation. Data were collected from the Turku University Hospital data lake.

The data collected at baseline and/or during follow‐up included: age, gender, date of allogeneic HSCT, R and D CMV serostatus, combined D/R CMV serostatus, donor type (matched unrelated, HLA identical sibling, and haploidentical), the primary reason for allogeneic HSCT (acute leukemias, myeloproliferative malignancies, lymphoproliferative malignancies, aplastic anemia), preconditioning regimen (myeloablative,^34^, ^35^ reduced intensity,^36^ and sequential^37^), stem cell source (peripheral blood, bone marrow), anti‐CMV medication (foscarnet, ganciclovir, and valganciclovir), CS‐CMV infection episode, number of outpatient visits, number and length of hospital admissions and intensive care, date and type of GVHD, and dates of CS‐CMV infection, relapse and death. Anti‐CMV medication, number of outpatient visits, number and length of hospital admissions, and intensive care were collected only from the first year of follow‐up. For further details of definitions and categorizations, see Supporting Information.

### 2.2 CMV‐serostatus analysis, CMV‐monitoring, CS‐CMV infection, and CS‐CMV infection episode

CMV‐serostatus was analyzed in donors and recipients by enzyme immunoassay according to the local laboratory procedure. After the transplantation, peripheral blood plasma CMV levels were monitored weekly for the first 60 days, and then every 2–4 weeks until the end of the first post‐transplant year by quantitative PCR using GeneProof CMV PCR Kit, 1 copy/ml corresponding to 1 IU/ml. The lower detection limit of the assay is 250 copies/ml, and a sample containing <50 copies/ml is reported as negative. CS‐CMV infection was defined as ≥1000 copies/ml, or the initiation of PET at any level of CMV DNAemia. PET was initiated with CMV DNA ≥1000 copies/ml by local protocol, or with CMV DNA levels <1000 copies/ml by clinical choice. CS‐CMV infection episode was defined as the occurrence of CS‐CMV infection, the start of any anti‐CMV medication, and the change of CMV DNAemia below the detection limit during anti‐CMV medication, followed by a period of two weeks with undetectable CMV nucleic acid levels in blood samples after cessation of anti‐CMV medication.

### 2.3 Outcome measures

The clinical outcomes included CMV seroprevalence, incidence and the number of CS‐CMV infections, time to the first CS‐CMV infection and CMV infection‐free survival, overall survival, relapse of the underlying malignancy and relapse‐free survival, acute and chronic GVHD and GVHD‐free survival.

Healthcare resource utilization outcomes included hospital readmission and intensive care unit admission rates, length of stay in the hospital and intensive care units, the number of outpatient hospital visits, and the use of anti‐CMV medication.

### 2.4 Statistical analyses

The continuous variables in the data were described using means and standard deviations if normally distributed and as medians along with lower and upper quartiles (Q_1_–Q_3_) if non‐normally distributed. The normality of the empirical distributions was tested using the Kolmogorov‐Smirnov test. Discrete variables were presented using observed frequencies and proportions.

Differences in patient characteristics (age, gender, recipient and donor serostatus, primary reason for allogeneic HSCT, preconditioning regimen, donor type, and HLA matching, and stem cell source) between patients with and without CS‐CMV infection were analyzed using Mann‐Whitney U‐test for numerical variables and Pearson's chi‐squared of Fisher exact test categorical attributes. The association of different patient characteristics and the occurrence of CS‐CMV infection (in general or multiple episodes) were analyzed using simple logistic regression.

Simple and multiple Cox proportional hazards models were used to analyze the overall survival of the patients. CMV infection‐free survival, relapse‐free survival, and GVHD‐free survival were analyzed using a simple and multiple proportional hazards model for the subdistribution of a competing risk because death or non‐relapse‐related death were considered competing risks. The Schoenfield residuals were checked to confirm the proportional hazards assumption, and if the assumption was not met, the Kaplan‐Meier estimator and log‐rank test were used for overall survival and the cumulative incidence function, and Gray's test for events with competing risks. Healthcare resource utilization due to CS‐CMV infection was further analyzed with a simple negative binomial model. The use of anti‐CMV medication between patients with one versus multiple infections as well as acute GVHD grade 0–2 versus 3–4 was analyzed using Pearson's chi‐squared or Fisher's exact test and the differences in total dosage were analyzed with Mann‐Whitney *U*‐test.

In all analyses where CS‐CMV infection or GVHD were used as independent variables, sensitivity analyses were performed and the model selection was based on the smallest value of the Akaike information criterion. For further details of the sensitivity analyses, see Supporting Information.

The results of the Cox proportional hazard models were presented using the average hazard ratios (HRs), and the results of logistic and negative binomial regression were presented as odds ratios (ORs) and incidence rate ratios (IRRs), respectively. The results of the log‐rank and Gray's test were reported using the chi‐squared test statistic together with the degrees of freedom. All results were presented with *p*‐values and corresponding 95% confidence intervals when applicable. *p*‐values less than 0.05 were considered statistically significant. Statistical analysis, tables, figures, and listings were all produced with R version 3.6.3 (R Core Team, 2018) using RStudio Server in Auria Clinical Informatics’ secure operating environment.

### 2.5 Ethical considerations

This study was approved by the Administration of the Hospital District of Southwest Finland (T94/2020) in the role of the data controller and was conducted in compliance with applicable national legislation. According to the Act of Secondary Use of Health and Social Data, informed consent is not required for studies based on patient records. MSD had oversight of the study. MSD had an initiative in the conception of the study, and MSD participated, together with other investigators, in the design of the study, and interpretation of the results, and MSD was responsible for the publication development. MSD did not participate in the data collection and analysis.

## 3 RESULTS

### 3.1 Baseline characteristics

Between January 1, 2013, and December 31, 2018, a total of 276 patients were identified. Twenty‐five patients were excluded from this study: 22 patients participated or had been considered for participation in the letermovir CMV reactivation prophylaxis study,^33^ one patient received syngeneic HSCT and two patients had not received HSCT. Altogether, 251 allogeneic HSCT patients were included in the study.

Baseline characteristics are presented in Table 1. The median age was 55 years, and the proportions of male and female patients were 56.2% and 43.8%, respectively. CMV seroprevalence was 69.7%. D/R CMV serostatus is detailed in Table 1. Thirty‐three (13.1%), 38 (15.1%), and 180 (71.7%) patients received a transplant from a haploidentical donor, HLA identical sibling donor, or matched unrelated donor, respectively. The reasons for allogeneic HSCT were acute leukemias (51.4%), lymphoproliferative diseases (29.5%), myeloproliferative diseases (15.9%), and (3.2%) aplastic anemia. Ninety‐seven, 90, and 64 patients received sequential, reduced intensity, or myeloablative preconditioning, respectively.

**TABLE 1:** Baseline characteristics of the study population

	All patients	No CMV infection	CMV infection	
	(*N* = 251)	(*N* = 103)	(*N* = 148)	*p*‐value
Age (yr), median (Q_1_–Q_3_)	55.0 (43.0, 62.0)	55.0 (38.5, 61.5)	55.0 (43.0, 62.0)	.401
Gender				.112
female	110 (43.8%)	39 (37.9 %)	71 (48.0 %)	
male	141 (56.2%)	64 (62.1 %)	77 (52.0 %)	
Recipient CMV serostatus				<.001
negative	76 (30.3%)	63 (61.2%)	13 (8.8 %)	
positive	175 (69.7%)	40 (38.8%)	135 (91.2 %)	
Donor CMV serostatus				<.001
negative	113 (45.0%)	66 (64.1 %)	47 (31.8 %)	
positive	138 (55.0%)	37 (35.9 %)	101 (68.2 %)	
D/R serostatus				<.001
D‐/R‐	58 (23.1%)	53 (51.5%)	5 (3.4%)	
D‐/R+	55 (21.9%)	13 (12.6%)	42 (28.4%)	
D+/R‐	18 (7.2%)	10 (9.7 %)	8 (5.4 %)	
D+/R+	120 (47.8%)	27 (26.2 %)	93 (62.8 %)	
Donor type and HLA matching				.074
matched unrelated	180 (71.7%)	76 (73.8 %)	104 (70.3 %)	
HLA identical sibling	38 (15.1%)	19 (18.4 %)	19 (12.8 %)	
haploidentical	33 (13.1%)	8 (7.8 %)	25 (16.9 %)	
Primary reason for allogeneic HSCT				.683
aplastic anemia	8 (3.2%)	2 (1.9%)	6 (4.1%)	
acute leukemias	129 (51.4%)	57 (55.3 %)	72 (48.6 %)	
lymphoproliferative diseases	74 (29.5%)	28 (27.2 %)	46 (31.1 %)	
myeloproliferative diseases	40 (15.9%)	16 (15.5 %)	24 (16.2 %)	
Type of preconditioning				.366
sequential	97 (38.6%)	45 (43.7 %)	52 (35.1 %)	
reduced intensity	90 (35.9%)	35 (34.0 %)	55 (37.2 %)	
myeloablative	64 (25.5%)	23 (22.3 %)	41 (27.7 %)	
Stem cell source				.049
peripheral blood	225 (89.6%)	97 (94.2 %)	128 (86.5 %)	
bone marrow	26 (10.4%)	6 (5.8 %)	20 (13.5 %)	

Acute leukemias included acute lymphoid leukemia, acute myeloid leukemia, and lymphoblastic lymphoma. Lymphoproliferative diseases included chronic lymphoid leukemia, non‐Hodgkin lymphoma, Hodgkin lymphoma, and multiple myeloma. Myeloproliferative diseases included myelodysplastic syndrome, myelofibrosis, and chronic myeloid leukemia. Differences between no CMV infection and CMV infection groups were tested using Mann‐Whitney U‐test age and the chi‐square test or Fisher's exact for other variables. CMV, cytomegalovirus; D, donor; R, recipient.

### 3.2 CMV infection

**FIGURE 1:**
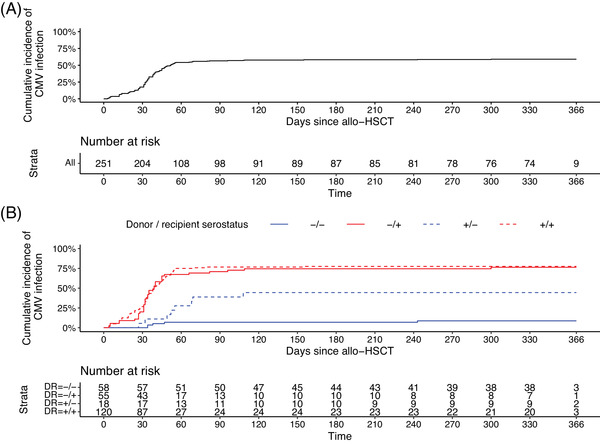
Cumulative incidence of CMV infection by one year after the allogeneic hematopoietic stem cell transplantation (HSCT) in (A) all patients and in (B) indicated by D/R status

One‐hundred forty‐six patients (58.1%) and 148 (59.0%) had CS‐CMV infection by week 24 and one year, respectively, and the median time to the first CS‐CMV infection was 34.5 (27.0, 45.0) days (Figure 1). CS‐CMV infection occurred in 77.1% and 17.1% of the seropositive and seronegative patients, respectively. One hundred twenty‐seven (85.8%) patients had one CS‐CMV infection episode, and 21 (14.2%) had ≥2 CS‐CMV infection episodes within one year (Table 2). One patient had the first CS‐CMV infection after one year.

**TABLE 2:** Clinical outcomes

	Overall (*n* = 251)	no CMV infection (*n* = 103)^a^	CMV infection (*n* = 148)^b^
CMV infection			
One infection (*n*, %)	127 (50.6 %)	n.a.	127 (85.8 %)
≥2 infections (*n*, %)	21 (8.4 %)	n.a.	21 (14.2 %)
Time to the first CMV infection (days)			
median (Q_1_–Q_3_)	34.5 (27.0, 45.0)	n.a.	34.5 (27.0, 45.0)
Acute GVHD (*n*, %)			
no acute GVHD	136 (54.2 %)	64 (62.1 %)	72 (48.6 %)
grade 1	16 (6.4 %)	6 (5.8 %)	10 (6.8 %)
grade 2	50 (19.9 %)	15 (14.6 %)	35 (23.6 %)
grade 3	24 (9.6 %)	8 (7.8 %)	16 (10.8 %)
grade 4	25 (10.0 %)	10 (9.7 %)	15 (10.1 %)
Chronic GVHD (*n*, %)			
grade 0‐1^#^	175 (69.7 %)	76 (73.8 %)	99 (66.9 %)
grade 2	33 (13.1 %)	13 (12.6 %	20 (13.5 %)
grade 3	43 (17.1 %)	14 (13.6 %)	29 (19.6 %)
Time to first GVHD (days)			
median (Q_1_–Q_3_)	51.0 (27.3, 87.8)	48.0 (27.0, 87.5)	52.0 (28.5, 85.5)
Relapse			
at week 24 (*n*, %)	36 (14.3 %)	14 (13.3 %)	22 (15.1 %)
at 1 year (*n*, %)	49 (19.5 %)	18 (17.5 %)	31 (20.9 %)
at 3 years (*n*, %)	75 (29.9 %)	27 (26.5 %)	48 (32.2 %)
All‐cause mortality			
at week 24 (*n*, %)	37 (14.7 %)	17 (16.2 %)	20 (13.7 %)
at 1 year (*n*, %)	62 (24.7%)	27 (26.2 %)	35 (23.6 %)
at 3 years (*n*, %)	96 (38.2 %)	35 (34.3 %)	61 (40.9 %)
Relapse mortality			
at week 24 (*n*, %)	8 (3.2 %)	3 (2.9 %)	5 (3.4 %)
at 1 year (*n*, %)	21 (8.4 %)	11 (10.7 %)	10 (6.8 %)
at 3 years (*n*, %)	42 (16.7 %)	17 (16.7 %)	25 (16.8 %)
Non‐relapse mortality			
at week 24 (*n*, %)	29 (11.6%)	14 (13.6%)	15 (10.1%)
at 1 year (*n*, %)	41 (16.3%)	16 (15.5%)	25 (16.9%)
at 3 years (*n*, %)	54 (21.5%)	18 (17.5%)	36 (24.3%)

Outcomes are reported at 1 year unless otherwise indicated (%).

^#^ includes patients without chronic GVHD and patients with mild grade 1 chronic GVHD.

^a^ The number of patients with no CMV infection at week 24 was 105.

^b^ The number of patients with CMV infection at week 24 was 146.

When analyzing the CMV‐free survival with the simple Cox proportional hazards model, D/R CMV serostatus was associated with the risk of CS‐CMV infection. Donor CMV serostatus did not influence on the risk of CS‐CMV infection in CMV seropositive recipients (D‐/R+ vs. D+/R+: HR 0.96, 95% confidence interval [CI] 0.67–1.39, *p* = .841) (Figure 1B and Table S1), but recipient CMV seronegativity was associated with lower risk for CS‐CMV infection (D+/R‐ vs. D+/R+: HR 0.35, 95% CI 0.18–0.66, *p* = .001) (Figure 1B and Table S1). The haploidentical graft was associated with higher CS‐CMV infection risk as compared to matched unrelated donor (HR 1.54, 95% CI 1.02–2.34, *p* < .001) (Figure S1A and Table S1). Other covariates, such as type of preconditioning, and the primary reason for allogeneic HSCT or GVHD, were not associated with differences in the CS‐CMV infection‐free survival.

In the multiple Cox proportional hazards model, donor CMV serostatus did not have effect on the risk of CS‐CMV infection in CMV seropositive recipients (D‐/R+ vs. D+/R+: HR 0.87, 95% CI 0.59–1.27, *p* = .463) (Table 3). Recipient CMV seronegativity was associated with a lower risk for CS‐CMV infection when the graft was received from a seropositive donor (D+/R‐ vs. D+/R+: HR 0.25, 95% CI 0.13–0.51, *p* < .001) (Table 3). Lymphoproliferative diseases, with borderline statistical significance, increased the CS‐CMV infection risk (HR 1.46, 95% CI 1.00–2.13, *p* = .050) (Table 3), even though the reason for allogeneic HSCT was not associated with CS‐CMV infection in the simple Cox proportional hazards model (Figure S1B and Table S1). A graft from an HLA identical sibling donor was associated with lower CS‐CMV infection risk (HR 0.55, 95% CI 0.34–0.88, *p* < .013) (Table 3). D‐/R‐ patients had a low risk of CS‐CMV infection in the simple (Figure 1B and Table S1) and multiple (Table 3) Cox proportional hazards models.

**TABLE 3:** Multiple Cox proportional hazards model of CMV infection in on post allogeneic hematopoietic stem cell transplantation (HSCT)

Covariate	Reference (if included as a categorical variable)	HR (95% CI)	*p*‐value
Age (year)		1.02 (0.99–1.03)	.635
Gender	Female	0.76 (0.55–1.06)	.105
D/R serostatus			
D‐/R‐	D+/R+	0.04 (0.02–0.11)	<.001
D‐/R+	D+/R+	0.87 (0.59–1.27)	.463
D+/R‐	D+/R+	0.25 (0.13–0.51)	<.001
Primary reason for allogeneic HSCT			
lymphoproliferative diseases^a^	acute leukemias^b^	1.46 (1.00–2.13)	.050
other^c^	acute leukemias^b^	1.02 (0.65–1.58)	.947
Type of preconditioning			
reduced intensity	sequential	1.30 (0.86–1.97)	.208
myeloablative	sequential	1.41 (0.89–2.25)	.142
Donor type and HLA matching			
HLA identical sibling	matched unrelated donor	0.55 (0.34–0.88)	.013
haploidentical	matched unrelated donor	1.17 (0.73‐1.88)	0.508
Stem cell source	peripheral blood	0.95 (0.57‐1.61)	0.868
GVHD^d^	no GVHD	0.86 (0.59–1.27)	.451

^a^ includes lymphoproliferative diseases (chronic lymphoid leukemia, non‐Hodgkin lymphoma, Hodgkin lymphoma) and multiple myeloma.

^b^ includes acute leukemias (acute lymphoid leukemia, acute myeloid leukemia, and lymphoblastic lymphoma).

^c^ includes myeloproliferative diseases (myelodysplastic syndrome, myelofibrosis, and chronic myeloid leukemia) and aplastic anemia. These patients were combined due to a low number of patients in disease groups.

^d^ based on the sensitivity analysis, GVHD was used as a binary covariate instead of a time‐varying covariate.

CI, confidence interval; CMV, cytomegalovirus; D, donor; GVHD, graft‐versus‐host disease; HSCT, hematopoietic stem cell transplantation; HR, hazards ratio; R, recipient.

Association of baseline and time‐dependent factors with ≥2 CS‐CMV infections during the first year after the transplantation was analyzed with a simple logistic regression. Factors associated with a higher risk for ≥2 CS‐CMV infections included myeloproliferative diseases and aplastic anemia as the primary reason for the allogeneic HSCT (OR 3.38, 95% CI 1.09–10.70, *p* = .034), presence of acute GVHD (OR 4.90, 95% CI 1.70–17.75, *p* = .006), and grade 2–3 chronic GVHD (OR 12.62, 95% CI 4.31–46.38, *p* < .001) (Table S2). Age was associated with ≥2 CS‐CMV infections but the effect size was small (Table S2).

### 3.3 CS‐CMV infection and mortality

**FIGURE 2:**
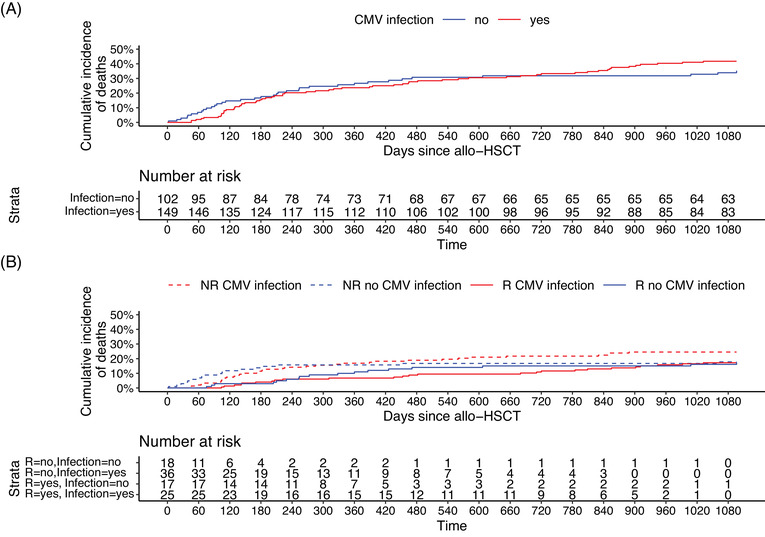
Cumulative incidence plots for (A) all‐cause mortality, and (B) relapse and non‐relapse mortality by 1‐year post allogeneic hematopoietic stem cell transplantation (HSCT). NR, no relapse; R, relapse

All‐cause mortality, relapse mortality, and non‐relapse mortality were investigated at one year and three years, and the mortality rates are detailed in Table 2. CS‐CMV infection did not increase the risk of all‐cause mortality at one year (HR 0.86, 95% CI 0.52–1.43, *p* = .569) or three years (HR 1.16, 95% CI 0.77–1.76, *p* = .485) (Figure 2A). Also, CMV infection did not increase the risk of relapse‐related deaths at one year ($X_{\left(1\right)}^{2}$ 1.17, *p* = .279) or three years (HR = 0.98, 95% CI 0.53–1.81, *p* = .938) of follow‐up (Figure 2B).

### 3.4 CS‐CMV infection, GVHD, and relapses

CS‐CMV infection did not increase the risk of GVHD at 24 weeks (HR 1.01, 95% CI 0.75–1.37, *p* = .936) or at one year (HR 1.00, 95% CI 0.73–1.35, *p* = .982). However, the risk of GVHD was higher in patients with ≥2 CS‐CMV infections compared to those without GVHD by one year (HR 1.61, 95% CI 1.03–2.51, *p* = .035) (Figure S2). In the multiple Cox proportional hazards model, GVHD risk was not higher in patients with one CS‐CMV infection (HR 1.06, 95% CI 0.74–1.53, *p* = .742), but the risk of GVHD was higher in patients with ≥2 CS‐CMV infections, (HR 1.65, 95% CI 1.02–2.66, *p* = .041) compared to those without CS‐CMV infection. CS‐CMV infection was not associated with relapses at one year (HR 1.21, 95% CI 0.68–2.16, *p* = .521) or three years (HR 1.23, 95% CI 0.77–1.98, *p* = .385).

### 3.5 CS‐CMV infection and healthcare resource utilization

In the study cohort, 208 (82.9%) patients were readmitted to the hospital. One hundred thirty‐three (89.8%) and 75 (72.8%) patients with and without CS‐CMV infection, respectively, were readmitted during the first year after the transplantation (Table 4). The number of hospital readmissions was higher in patients with CS‐CMV infection compared to those without CS‐CMV infection [median (Q_1_–Q_3_) 3.0 (2.0–5.0) vs. 2.0 (1.0–3.5), IRR 1.38, 95% CI 1.10–1.73, *p* = .005]. Correspondingly, the number of hospital readmissions was higher in patients with ≥2 CMV infections compared to those without CMV infection [median (Q_1_–Q_3_) 6.0 (3.0–7.0) vs. 2.0 (1.00–3.5); IRR 2.15, 95% CI 1.53–3.03, *p* < .001] (Table 4).

**TABLE 4:** Healthcare resource utilization in one year post allogeneic hematopoietic stem cell transplantation (HSCT)

	All patients	No CMV infection	one CMV infection	≥2 CMV infections	
	(*N* = 251)	(*N* = 103)	(*N* = 127)	(*N* = 21)	*p*‐value
Number of patients with rehospitalization^#^					<.001
(*n*, %)	208 (82.9 %)	75 (72.8 %)	112 (88.2 %)	21 (100.0 %)	
Number of hospital readmissions					<.001
median (Q_1_–Q_3_)	3.0 (2.0, 5.0)	2.0 (1.0, 3.5)	3.0 (2.0, 5.0)	6.0 (3.0, 7.0)	
Total length of hospital stay (days)					<.001
median (Q_1_–Q_3_)	24.5 (9.75, 56.0)	14.0 (4.0, 37.0)	27.0 (14.8, 53.0)	75.0 (56.0, 92.0)	
Number of patients in intensive care unit^#^					.314
(*n*, %)	24 (9.6 %)	7 (6.8 %)	16 (12.6 %)	1 (4.8 %)	
Length of stay in the intensive care unit (days)					.356
median (Q_1_–Q_3_)	3.0 (2.0, 6.0)	2.0 (2.0, 3.0)	4.0 (2.0, 6.0)	3.0 (3.0, 3.0)	
Number of hospital outpatient visits					.964
median (Q_1_–Q_3_)	21.0 (15.0, 30.0)	22.0 (15.0, 30.0)	21.0 (15.0, 29.0)	20.0 (15.0, 33.0)	

Differences between no CMV infection, one CMV infection, and ≥2 CMV infections were tested using the Kruskal‐Wallis test. # Differences between no CMV infection, one CMV infection, and ≥2 CMV infections were tested with the chi‐square test or Fisher's exact test. CMV, cytomegalovirus; HSCT, hematopoietic stem cell transplantation.

The additional length of hospital stay was longer in patients with CS‐CMV infection compared to those without CMV infection [median days (Q_1_–Q_3_) 27.0 (14.8–53.0) vs. 14.0 (4.0–37.0); IRR 1.48, 95% CI 1.12–1.94, *p* = .005]. Also, patients with ≥2 CS‐CMV had longer additional length of hospital stay [median days (Q_1_–Q_3_) 75.0 (56.0–92.0) vs. 14.0 (4.0–37.0); IRR 2.71, 95% CI 1.76–4.35, *p* < .001].

CS‐CMV infection was not associated with increased intensive care admission (Table 4) or its length. We did not see differences in the number of outpatient visits, either (Table 4).

One hundred forty‐eight patients had CS‐CMV infection, and 145 (98.0%) patients received PET. One hundred twenty‐four patients received PET for one CMV infection, and 21 patients received PET for ≥2 CMV infections (Table S3).

Frequencies and the medians of total doses for all anti‐CMV drugs are detailed in Table S3. In patients with CS‐CMV infection, 73.0% and 62.2% received valganciclovir and foscarnet, respectively. Ganciclovir (36.5%) was less often used.

Foscarnet was used more often in patients with ≥2 CS‐CMV infections compared to patients with one CS‐CMV infection (90.5% and 57.5%, respectively, *p* = .004) (Table S3). There was no difference in the proportions of the patients with one CS‐CMV infection versus ≥2 CS‐CMV infection episodes who received ganciclovir or valganciclovir. The total doses did not differ between anti‐CMV drugs (Table S3).

Patients with grade 3–4 acute GVHD received more often foscarnet (51.0% vs. 33.2%, *p* = .020), and the total dose of foscarnet was higher compared to the patients with grade ≤2 acute GVHD (Table S3). There was no difference in the frequencies or total doses of ganciclovir or valganciclovir between patients with grade ≤2 or grade 3–4 acute GVHD (Table S3).

## 4 DISCUSSION

This retrospective single‐center study investigated clinical and healthcare burdens due to CS‐CMV infection in allogeneic HSCT patients by one year after allogeneic HSCT, and mortality rate up to three years. This is the first study reporting CMV infection rate and CMV seroprevalence in Finnish allogeneic HSCT patients. CMV seroprevalence was 69.7%, and CS‐CMV infection occurred in 59.0% of all patients, and 77.1% of the CMV seropositive patients. Also, 14.2% of the patients with CS‐CMV infection experienced ≥2‐CMV infections during the first year after the transplantation. CS‐CMV infection was associated with greater healthcare resource utilization, indicated by higher readmission rates and longer length of hospital stay.

D/R CMV serostatus has a significant impact on the risk of CMV infection and clinical outcomes after allogeneic HSCT.^2^, ^4^, ^5^ Recipient seropositivity is associated with CMV‐related outcomes, such as delayed CMV‐specific immune reconstitution, repeated CMV infections, additional anti‐CMV treatment courses, late CMV infection, and the development of CMV disease.^3^, ^17^, ^18^, ^19^, ^20^, ^21^ In this study, recipient CMV seropositivity was associated with CS‐CMV infection, and D+/R‐ and D‐/R‐ patients had lower CS‐CMV infection rates in the 1‐year follow‐up, and these findings are in line with the published literature. Other risk factors include high‐dose corticosteroids, T‐cell depletion, acute and chronic GVHD, and the use of mismatched or unrelated donors.^8^, ^15^, ^21^, ^22^, ^23^, ^24^, ^25^ In our study, donor type and HLA matching, and the primary reason for allogeneic HSCT had independent effects on the CMV infection risk. We also investigated the rate of subsequent CMV infections, and ≥2 CMV infections occurred in 14% of the study patients. In a recent study, 23% of all seropositive allogeneic HSCT recipients and 31% of those who received PET for their first CMV reactivation, had ≥2 CMV reactivations during the first year after transplantation.^6^

CMV seroprevalence was 69.7% in the study cohort. Observed CMV seroprevalence is in line with the CMV seroprevalence reported from the Finnish biobank cohort of pregnant women, with CMV seroprevalence of 84.5% in 1992 and 71.5% in 2012.^32^ Of note, the population in our study covers approximately 40% of the allogeneic HSCT patients during the study period and represents Finnish population in the limited geographical regions. Also, the population in our study was older than pregnant women typically. These factors should be considered when extrapolating the observed CMV seroprevalence to all allogeneic HSCT patients in Finland.

CMV seropositivity and CMV infection have been reported to associate with negative clinical outcomes in allogeneic HSCT patients.^2^, ^4^, ^5^ In the previous studies, positive donor or recipient CMV serostatus were associated with lower survival among allogeneic HSCT recipients.^38^, ^39^ In a large retrospective study, positive donor or recipient CMV serostatus was associated with lower overall survival acute myeloid leukemia and acute lymphoblastic leukemia patients,^4^ and CMV infection has been associated with higher non‐relapse mortality contributing to lower overall survival.^4^, ^7^, ^40^ PET has reduced the incidence of CMV disease and CMV disease‐related mortality.^4^, ^5^, ^15^, ^16^ However, CMV replication at any level has been reported to associate with higher mortality even after adjusting the use of PET.^5^ A post‐hoc analysis of the phase 3 trial investigating a 100‐day CMV prophylaxis with letermovir in CMV seropositive recipients suggested that all‐cause mortality by week 48 was lower in the letermovir group (12%) compared to that in the placebo group (17%) and the difference was associated with a decrease in CMV reactivation‐related mortality in letermovir group, despite of PET.^41^ In our study, mortality rates were 14.7%, 24.7%, and 38.2% at week 24, one year, and three years, respectively, and we did not see the difference in mortality rates among patients with or without CS‐CMV infection. Also, relapse mortality was not associated with CS‐CMV infection. However, looking at the mortality curves, one may see higher early all‐cause and non‐relapse mortality among patients without CMV infection. The mortality follow‐up was started from the transplantation. We retrospectively grouped the patients by CS‐CMV infection, which is a time‐related event, and the mortality analyses may contain an inherent bias due to the clustering of early transplantation‐related deaths in the group of patients without CS‐CMV infection. Green et al.^5^ analyzed the association of CMV infection at day 0–60 after transplantation with the mortality by one year among day 100 survivors. This landmark analysis suggested that CMV reactivation with high viral loads was associated with all‐cause and non‐relapse mortality even if the early transplantation‐related mortality had been excluded.^5^

Previous studies report that D+/R+ serology is associated with a lower incidence of acute GVHD in chronic myeloid leukemia, whereas D/R serology or CMV infection is not associated with acute GVHD in other diseases.^4^ We did not see the difference between the CS‐CMV infection and the incidence of GVHD. Also, the incidence of CS‐CMV infection did not differ in patients with grade 3–4 acute GVHD compared with those with grade ≤2 acute GVHD, but the association between ≥2 CMV infections and GVHD remained significant after multiple adjustments. From this data, it is difficult to say the effect of ≥2 CMV infections on the incidence of GVHD, because CMV infection and GVHD often coincide, and we did not carry out a competing event analysis here. Previously, it has been reported that patients with acute GVHD and no CMV infection had a long time to fatal outcome compared with the patients hospitalized with or because of CMV infection.^31^

In our study, the malignancy relapse rate was 19.5% and 29.9% by one year and three years, respectively, and CS‐CMV infection was not associated with relapse. CMV infection has been postulated to reduce relapses in certain hematological malignancies, possibly by activating the graft and tumor immunity,^42^, ^43^ and a recent meta‐analysis suggested a lower risk of acute myeloid leukemia relapse in patients with CMV infection.^44^ A large retrospective study, however, did not find an association between CMV infection and relapses.^4^

Healthcare costs due to CMV infection have been compared with costs in patients with acute GVHD.^31^ CMV infection was associated with higher hospital readmission rates, a longer length of hospital stay, and increased total costs.^31^ Patients hospitalized with CMV infection received more often foscarnet and cidofovir, contributing to the higher pharmacy costs.^31^ Another study showed that CMV infection and PET were associated with a longer length of stay for HSCT procedures, a higher number of hospital readmissions, a longer total length of hospital stay, and higher inpatient costs. More detailed analysis suggested that higher inpatient costs originated from hospital facility and procedure costs, laboratory services, and pharmacy costs.^45^ Two retrospective registry‐based studies in the United States showed that CMV infection caused increased healthcare resource use already within 100 days^29^ and by one year post allogeneic HSCT.^30^ In our study, CS‐CMV infection was associated with a higher readmission rate and longer length of hospital stay. When CS‐CMV infection is observed, patients are admitted to the hospital, and PET is initiated with intravenously administered ganciclovir or foscarnet, which in our study may contribute to the observed higher hospitalization rate of patients with CS‐CMV infection. When clinical improvement is observed, PET is continued after hospital discharge either with intravenously administered ganciclovir or foscarnet in the outpatient clinic, or with per os valganciclovir, and this is seen as a relatively high proportion of patients receiving valganciclovir. Patients with ≥2 CMV infections received more often foscarnet, but there was no difference in the proportions of the patients receiving other anti‐CMV drugs with respect to the number of CMV infections.

In contrast, a single‐center retrospective study investigating costs in 310 allogeneic HSCT patients did not report an association between CMV infection and changes in healthcare costs.^28^ Studies investigating costs, as our study also, often are based on retrospective data, which may contain bias difficult to control. Differences in healthcare and reimbursement policies inevitably impact cost formation, and therefore studies reporting costs must be interpreted with caution. Yet, analysis of the healthcare resource utilization in the prospective 100‐day CMV prophylaxis trial with letermovir suggested that a lower CMV reactivation rate with letermovir prophylaxis was associated with fewer readmissions and shorter total readmission length of stay at weeks 14, 28, and 48.^46^

Allogeneic HSCT patients report a negative impact on the quality of life in many dimensions of quality‐of‐life questionnaires, including physical, psychological, social, and role functions, especially during the first year after the transplantation.^47^ The quality of life is lower during the transplantation inpatient stay and gradually improves during the first post‐procedure year after hospital discharge.^48^ Impacted dimensions of the quality of life during the inpatient stay included physical functioning, pain, appetite, and sleep among others.^48^ Although not reported in the cited study and not investigated in our study, one may presume that hospital readmission during the first year after the transplantation has a negative impact on the quality of life.

In conclusion, CMV seroprevalence is relatively high among allogeneic HSCT patients in Finland. Clinically significant CMV infection was common, occurred within three months after the transplantation, and repeated CMV infection was associated with GVHD. CMV infection was associated with a higher hospital readmission rate and longer length of hospital stay.

## CONFLICT OF INTEREST

JR received the Principal Investigator fee, and Turku University Hospital District received institutional research funding for this study. RK is an employee of MSD Finland Oy and owns stocks of Merck & Co., Inc., Rahway, NJ, USA.

## Supporting information



Supporting information

Supporting information

Graphical Abstract
