# Supplementary material for: Clinical and healthcare burden of disease associated with cytomegalovirus in allogeneic hematopoietic stem cell transplantation – A retrospective single‐center study
Source: Transpl Infect Dis. 2022 Sep 27;24(6):e13947. doi: 10.1111/tid.13947 (PMC10369922; doi:10.1111/tid.13947)
Supplement: Supplementary file 1 — Supporting information [file TID-24-e13947-s002.docx]

**Figure S1.** Cumulative incidence CMV infection by (A) donor type and HLA matching, and (B) the reasons for allogeneic HSCT in 1 year post allogeneic HSCT. The reasons for allogeneic HSCT were grouped as follows: lymphoproliferative diseases (chronic lymphoid leukemia, non-Hodgkin lymphoma, Hodgkin lymphoma) and multiple myeloma; acute leukemias (acute lymphoid leukemia, acute myeloid leukemia and lymphoblastic lymphoma); myeloproliferative diseases (myelodysplastic syndrome, myelofibrosis and chronic myeloid leukemia) and aplastic anemia. Cumulative incidence of CMV infection was analysed with a simple Cox proportional hazards model with death as a competing event. HLA, HLA identical sibling donor; matched, matched unrelated donor.

A.


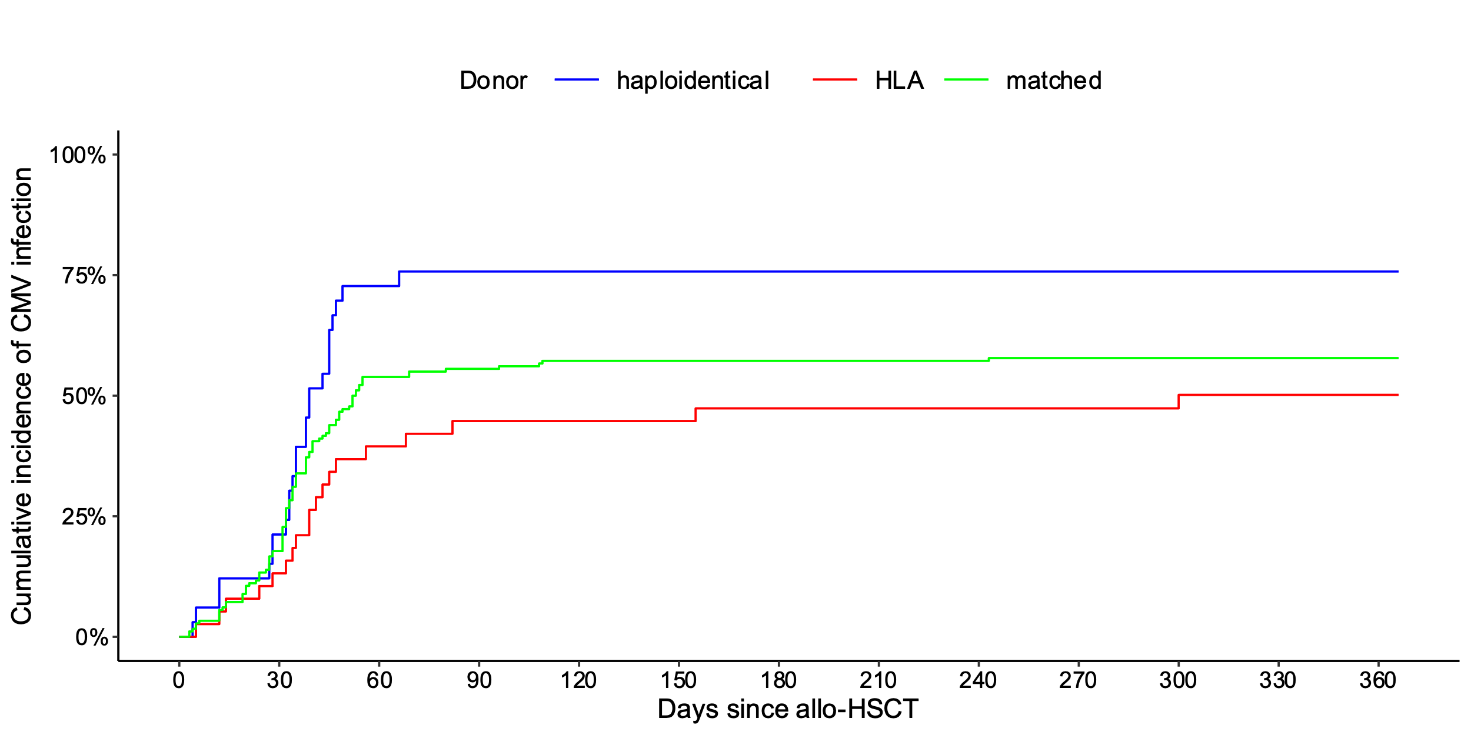


B.


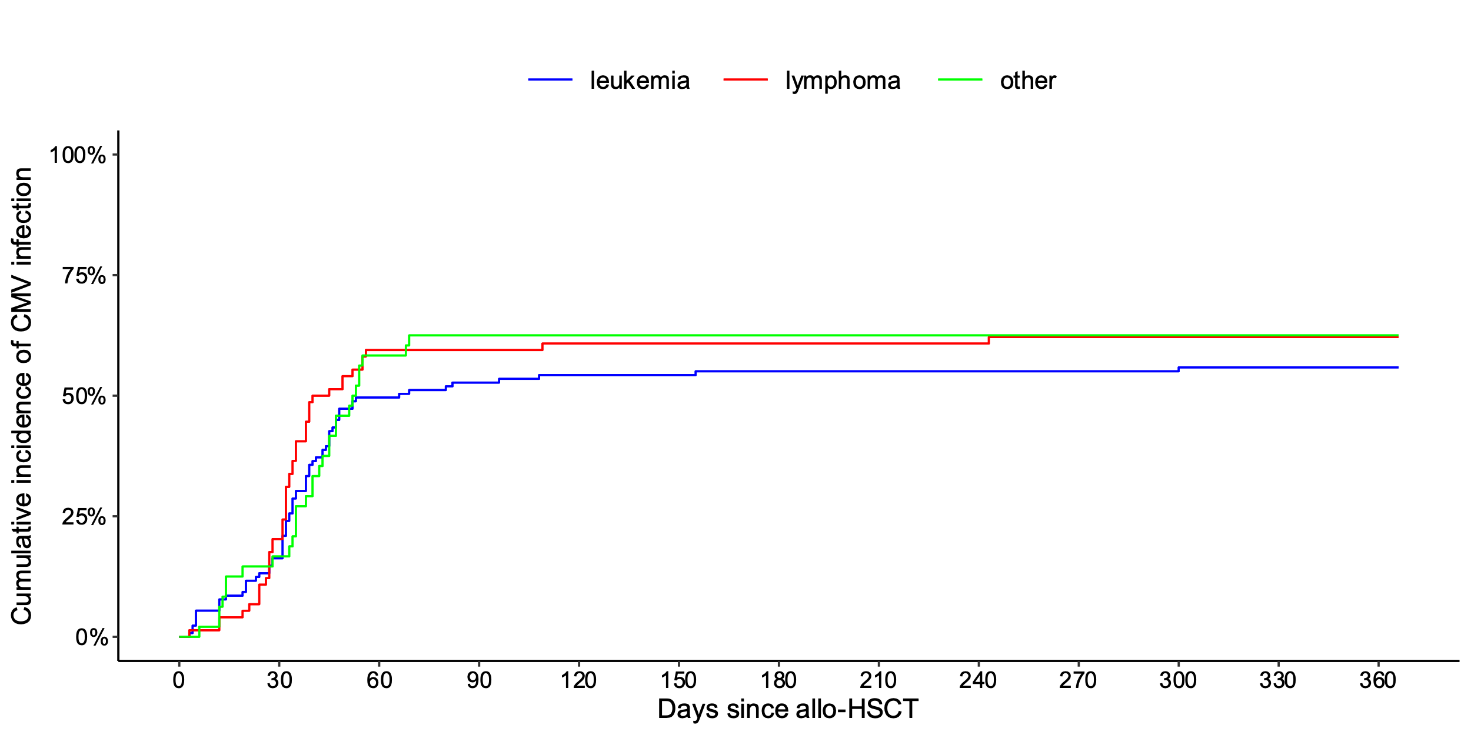


**Figure S2**. Cumulative incidence plots for GVHD in patients without CMV infection, with one CMV infection, and with ≥2 CMV infections by 1 year post allogeneic HSCT.


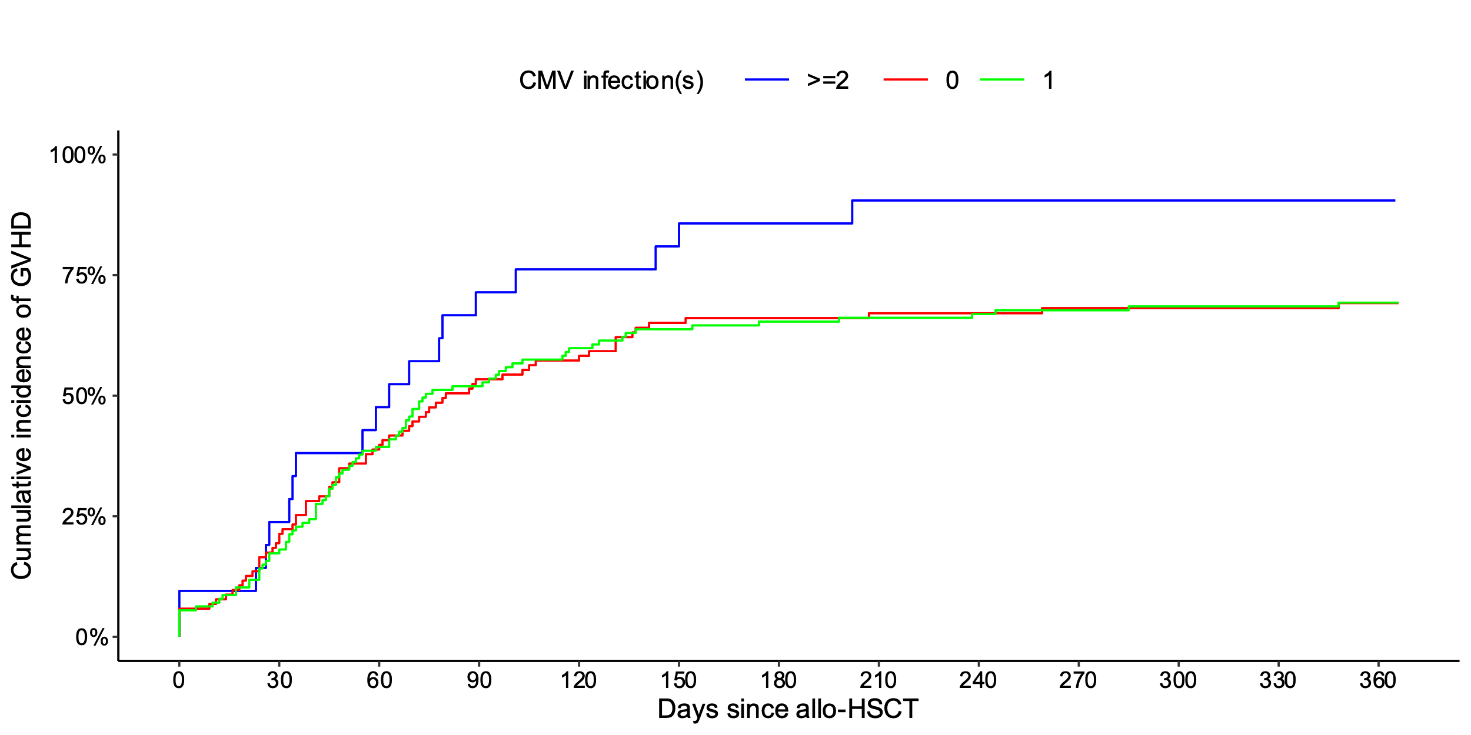


| Table S1. Simple proportional hazards analysis of CMV infection in 1 year post allogenic HSCT | | | | | |
| --- | --- | --- | --- | --- | --- |
| Covariate | reference (if included as categorical variable) | HR | CI 95% lower | CI 95% upper | p-value |
| Age (yr) |  | 1.01 | 0.99 | 1.02 | 0.357 |
| Gender | female | 0.770 | 0.56 | 1.06 | 0.107 |
| Recipient CMV positive | negative | 8.08 | 4.66 | 14.01 | <0.001 |
| Donor CMV positive | negative | 2.31 | 1.63 | 3.26 | <0.001 |
| D/R serostatus | | | | | |
| D-/R- | D+/R+ | 0.06 | 0.02 | 0.15 | <0.001 |
| D-/R+ | D+/R+ | 0.96 | 0.67 | 1.39 | 0.841 |
| D+/R- | D+/R+ | 0.35 | 0.18 | 0.66 | 0.001 |
| The primary reason for allogeneic HSCT | | | | | |
| lymphoproliferative diseases^a^ | acute leukemias^b^ | 1.21 | 0.84 | 1.76 | 0.306 |
| other^c^ | acute leukemias^b^ | 1.11 | 0.74 | 1.67 | 0.600 |
| Type of preconditioning | | | | | |
| reduced intensity | sequential | 1.26 | 0.87 | 1.83 | 0.224 |
| myeloablative | sequential | 1.36 | 0.91 | 2.03 | 0.136 |
| Donor type and HLA matching | | | | | |
| HLA identical sibling | matched unrelated donor | 0.76 | 0.47 | 1.21 | 0.244 |
| haploidentical | matched unrelated donor | 1.54 | 1.02 | 2.06 | 0.040 |
| Stem cell source | peripheral blood | 1.52 | 1.00 | 2.32 | 0.052 |
| Acute GVHD | no acute GVHD | 1.31 | 0.96 | 1.80 | 0.094 |
| Chronic GVHD grade 2-3 | grade ≤1 | 1.13 | 0.81 | 1.56 | 0.476 |
| a, includes lymphoproliferative diseases (chronic lymphoid leukemia, non-Hodgkin lymphoma, Hodgkin lymphoma) and multiple myeloma.  b, includes acute leukemias (acute lymphoid leukemia, acute myeloid leukemia and lymphoblastic lymphoma).  c, includes myeloproliferative diseases (myelodysplastic syndrome, myelofibrosis and chronic myeloid leukemia) and aplastic anemia. These patient groups were combined due to low number of patients in disease groups. CI, confidence interval; CMV, cytomegalovirus; D, donor; GVHD, graft-versus-host disease; HSCT, hematopoietic stem cell transplantation; OR, odds ratio; R, recipient | | | | | |

| Table S2. Simple logistic regression analysis of ≥2 vs. 1 CMV infection in 1 year post allogenic HSCT | | | | | |
| --- | --- | --- | --- | --- | --- |
| Covariate | reference (if included as categorical variable) | OR | CI 95% lower | CI 95% upper | p-value |
| Age (yr) |  | 1.05 | 1.00 | 1.19 | 0.046 |
| Gender | female | 0.65 | 0.25 | 1.64 | 0.366 |
| Recipient CMV positive | negative | 0.90 | 0.22 | 6.11 | 0.897 |
| Donor CMV positive | negative | 3.18 | 1.01 | 14.10 | 0.075 |
| D/R serostatus ^d^ |  |  |  |  |  |
| D-/R- | D+/R+ | n.a. | n.a | n.a. | n.a. |
| D-/R+ | D+/R+ | 0.37 | 0.08 | 1.19 | 0.132 |
| D+/R- | D+/R+ | 1.60 | 0.22 | 7.72 | 0.583 |
| The primary reason for allogeneic HSCT | | | | | |
| lymphoproliferative diseases^a^ | acute leukemias^b^ | 1.39 | 0.42 | 4.48 | 0.575 |
| other^c^ | acute leukemias^b^ | 3.38 | 1.09 | 10.70 | 0.034 |
| Type of preconditioning | | | | | |
| reduced intensity | sequential | 1.50 | 0.50 | 4.80 | 0.474 |
| myeloablative | sequential | 1.31 | 0.38 | 4.54 | 0.660 |
| Donor type and HLA matching | | | | | |
| HLA identical sibling | matched unrelated donor | 0.60 | 0.09 | 2.37 | 0.522 |
| haploidentical | matched unrelated donor | 0.45 | 0.07 | 1.71 | 0.301 |
| Stem cell source | peripheral blood | 1.08 | 0.24 | 3.63 | 0.911 |
| Acute GVHD | no acute GVHD | 4.90 | 1.70 | 17.75 | 0.006 |
| Chronic GVHD grade 2-3 | grade ≤1 | 12.62 | 4.31 | 46.38 | <0.001 |
| a, includes lymphoproliferative diseases (chronic lymphoid leukemia, non-Hodgkin lymphoma, Hodgkin lymphoma) and multiple myeloma.  b, includes acute leukemias (acute lymphoid leukemia, acute myeloid leukemia and lymphoblastic lymphoma).  c, includes myeloproliferative diseases (myelodysplastic syndrome, myelofibrosis and chronic myeloid leukemia) and aplastic anemia. These patient groups were combined due to low number of patients in disease groups.  d, D+/R+ n=16, D-/R+ n=3, D+/R- n=2. D-/R- n=0, OR, 95% CI was not analyzed.  CI, confidence interval; CMV, cytomegalovirus; D, donor; GVHD, graft-versus-host disease; HSCT, hematopoietic stem cell transplantation; n.a., not applicable; OR, odds ratio; R, recipient | | | | | |

| Table S3. Use of anti-CMV medication | | | | | | | |
| --- | --- | --- | --- | --- | --- | --- | --- |
|  | overall (n=148) | 1 infection (n=127) | ≥2 infections (n=21) | p-value | acute GVHD,  gr ≤2 (n=202) | acute GVHD,  gr 3-4 (n=49) | p-value |
| not treated (n, %) | 3 (2.0%) | 3 (2.4%) | 0 (0.0%) | n.a. | 87 (43.1%) | 19 (38.8%) | n.a. |
| Ganciclovir (n, %)^#^ | 54 (36.5 %) | 47 (37.0 %) | 7 (33.3 %) | 0.746 | 42 (28.8%) | 12 (24.5%) | 0.572 |
| median, mg (Q_1_-Q_3_) | 3700 (1410, 7290) | 3490 (1480, 6950) | 6000 (900, 10500) | 0.747 | 3600 (1290, 7290) | 3850 (1950, 6380) | 0.819 |
| Valganciclovir, (n, %)^#^ | 108 (73.0%) | 93 (73.2 %) | 15 (71.4 %) | 0.863 | 89 (44.1%) | 19 (38.8%) | 0.503 |
| median, mg (Q_1_-Q_3_) | 36000 (20300, 61400) | 34200 (20700, 64800) | 43200 (19400, 58100) | 0.954 | 35100 (20700, 69300) | 43200 (19400, 45900) | 0.333 |
| Foscarnet (n, %)^#^ | 92 (62.2%) | 73 (57.5 %) | 19 (90.5 %) | 0.004 | 67 (33.2%) | 25 (51.0%) | 0.020 |
| median, mg (Q_1_-Q_3_) | 149000 (52800, 283000) | 148000 (54000, 256000) | 195000 (73600, 308000) | 0.455 | 112000 (40200, 255000) | 195000 (120000, 350000) | 0.045 |
| Drug doses are presented as total dose per patient. Differences between total dosages were tested with Mann-Whitney U-test. # Differences between proportions were tested with two-proportions z-test. CMV, cytomegalovirus; gr, grade; GVHD, graft-versus-host disease; n.a., not applicable. | | | | | | | |
