# Supplementary material for: Clinical and healthcare burden of disease associated with cytomegalovirus in allogeneic hematopoietic stem cell transplantation – A retrospective single‐center study
Source: Transpl Infect Dis. 2022 Sep 27;24(6):e13947. doi: 10.1111/tid.13947 (PMC10369922; doi:10.1111/tid.13947)
Supplement: Supplementary file 2 — Supporting information [file TID-24-e13947-s001.docx]

**Supplementary materials**

**Conditioning, GVHD-prophylaxis and post-HSCT follow-up**

In this study, data on the type of conditioning treatment, as well as on the appearance acute or chronic GVHD, or disease relapse was collected as indicated in the electronic patient data. Detailed, patient-level on conditioning regimen, GVHD prophylaxis or relapse surveillance was out-of-scope. Below, conditioning regimens and GVHD prophylaxis are described at general level, and variations at patient level may exist.

The reduced intensity conditioning (RIC) regimens used during the study period mainly comprised of fludarabine combined with two-days busulfan (FluBu2) or treosulfan 10 mg/m^2^ intravenously for three days^1^. The most used myeloablative conditioning regimens included fludarabine in combination with four-days busulfan or combination of busulfan either with high-dose cyclophosphamide or 12 Gy total body irradiation^2^. In the haploidentical donor setting the most used MAC-regimen was the combination of thiothepa, fludarabine and busulfan ^3^. Sequential conditioning consisted of an induction with a combination of fludarabine, high-dose cytarabine and idarubicin or amsacrine, followed by a conditioning with cyclophosphamide (80-120 mg/kg) and TBI 4 Gy (FLAMSA-RIC) ^4^.

During the study period, the GVHD prophylaxis in case of HLA-identical sibling or matched unrelated donor consisted of a short course of methotrexate, rabbit-derived anti-thymocyte globulin (ATG; Thymoglobulin®, Sanofi Genzyme) 2.5 mg/kg on days -2 and -1, calcineurin inhibitor tacrolimus and mycophenolate mofetil on days 1-30. In haploidentical transplantations, GVHD prophylaxis included post-transplant high-dose cyclophosphamide (50mg/kg on days +3 and +4), mycophenolate mofetil and tacrolimus during the study period.

After allogeneic HSCT, the patients were actively monitored for disease relapse according to local protocols. Diagnosis and grading of acute GVHD and chronic GVHD were based on clinical and histopathological findings ^5,6^.

**Categorization of the primary reasons for allogeneic HSCT**

The patients were categorized into four groups by the underlying malignancy. Due to the low number of severe aplastic anemia patients, patients with myeloproliferative diseases and aplastic anemia were combined in further analyses, named as “other”.

| Categorization of the primary reasons for allogeneic HSCT | |
| --- | --- |
| Acute leukemias | acute myeloid leukemia, acute lymphoblastic leukemia and lymphoblastic lymphoma |
| Lymphoproliferative diseases | Hodgkin lymphoma, non-Hodgkin lymphoma, multiple myeloma and chronic lymphocytic leukemia |
| Myeloproliferative diseases | myelodysplastic syndrome, chronic myelomonocytic leukemia, chronic myeloid leukemia and myelofibrosis |
| Aplastic anemia | severe aplastic anemia |

**Definitions of Healthcare resource utilization outcomes**

Definitions of the Healthcare resource utilization outcomes are listed in the table below.

| Definitions of the Healthcare resource utilization outcomes | |
| --- | --- |
| Outcome of Interest | Definition |
| Number of hospital readmissions | Number of hospital admissions after the initial hospital stay for allogeneic HSCT procedure. Readmission must be preceded by hospital discharge indicated in the electronic hospital administrative operational data. |
| Additional hospital length of stay | Cumulative number of hospital days up to 1 year post allo-HSCT, excluding initial allogeneic HSCT procedure hospital days |
| Intensive care unit patients (n, %) | Proportion of patients admitted to intensive care unit post allogeneic HSCT procedure up to 1 year. |
| Length of stay in the intensive care unit | Cumulative number of intensive care unit days up to 1 year post allogeneic HSCT |
| Number of out-patient hospital visits | Number of out-patient visits in the hospital for any reason up to 1 year post allogeneic HSCT |
| Use of anti-CMV drugs (total drug dose consumption: ganciclovir, valganciclovir, foscarnet, cidofovir) | Total consumption of anti-CMV medication during the study period in the study cohort receiving anti-CMV medication. |
| CMV, cytomegalovirus; GVHD, graft-versus-host-disease; HSCT, hematopoietic stem cell transplantation; PET, preemptive therapy | |

**Sensitivity analysis**

CMV infection:

1) no/yes
2) 0/1/≥2

GVHD:

Preliminary: Cramer von Mises test for time-invariant effects in multiple proportional hazards model for the subdistribution of a competing risk. Based on test results, either included in the model as a time-varying effect or time-invariant categorized indicator.

1) no/yes (regardless of type and grade)

2) acute no/yes (regardless of grade) + chronic 0-1/2-3
3) acute grade 0-2/3-4 + chronic grade 0-1/2-3

4) acute grade 0/1/2/3/4 + chronic grade 0-1/2/3

The model selection of categorized indicator was based on the Akaike information criterion and the model with the smallest value and meeting the model assumptions was selected.

**References:**

1. Reshef R, Porter DL. Reduced-intensity conditioned allogeneic SCT in adults with AML. *Bone Marrow Transplant.* 2015;50(6):759-769.

2. Gooptu M, Kim HT, Ho VT, et al. A Comparison of the Myeloablative Conditioning Regimen Fludarabine/Busulfan with Cyclophosphamide/Total Body Irradiation, for Allogeneic Stem Cell Transplantation in the Modern Era: A Cohort Analysis. *Biol Blood Marrow Transplant.* 2018;24(8):1733-1740.

3. Duléry R, Bastos J, Paviglianiti A, et al. Thiotepa, Busulfan, and Fludarabine Conditioning Regimen in T Cell-Replete HLA-Haploidentical Hematopoietic Stem Cell Transplantation. *Biol Blood Marrow Transplant.* 2019;25(7):1407-1415.

4. Rodríguez-Arbolí E, Labopin M, Tischer J, et al. FLAMSA-Based Reduced-Intensity Conditioning versus Myeloablative Conditioning in Younger Patients with Relapsed/Refractory Acute Myeloid Leukemia with Active Disease at the Time of Allogeneic Stem Cell Transplantation: An Analysis from the Acute Leukemia Working Party of the European Society for Blood and Marrow Transplantation. *Biol Blood Marrow Transplant.* 2020;26(11):2165-2173.

5. Przepiorka D, Weisdorf D, Martin P, et al. 1994 Consensus Conference on Acute GVHD Grading. *Bone Marrow Transplant.* 1995;15(6):825-828.

6 Jagasia MH, Greinix HT, Arora M, et al. National Institutes of Health Consensus Development Project on Criteria for Clinical Trials in Chronic Graft-versus-Host Disease: I. The 2014 Diagnosis and Staging Working Group Report. *Biol Blood Marrow Transplant.* 2015;21(3):389-401.
