# Supplementary material for: Clinical and healthcare burden of disease associated with cytomegalovirus in allogeneic hematopoietic stem cell transplantation – A retrospective single‐center study
Source: Transpl Infect Dis. 2022 Sep 27;24(6):e13947. doi: 10.1111/tid.13947 (PMC10369922; doi:10.1111/tid.13947)
Supplement: Supplementary file 3 — Graphical Abstract [file TID-24-e13947-s003.pdf]

Clinical and healthcare burden of disease associated with cytomegalovirus in allogeneic hematopoietic stem cell transplantation – a retrospective single-center study

@TheTxIDJournal

Ranti J *et al*. ***Transplant Infectious Diseases***. 2022.

Study cohort

Turku University Hospital electronic patient data

All allogeneic HSCT patients in 2013-2018

Study cohort
- 251 patients
- follow-up 3 years

Key finding: CMV infection was common

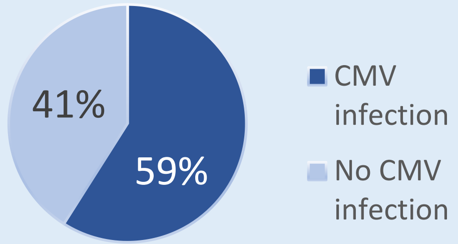

14% had ≥2 CMV infections

CMV INFECTION BY D/R STATUS

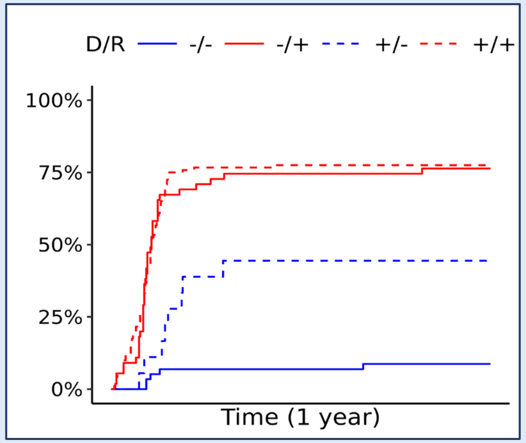

Key finding: CMV was associated with higher hospital resource use

ADDITIONAL HOSPITAL DAYS AFTER TRANSPLANTATION BY 1 YEAR

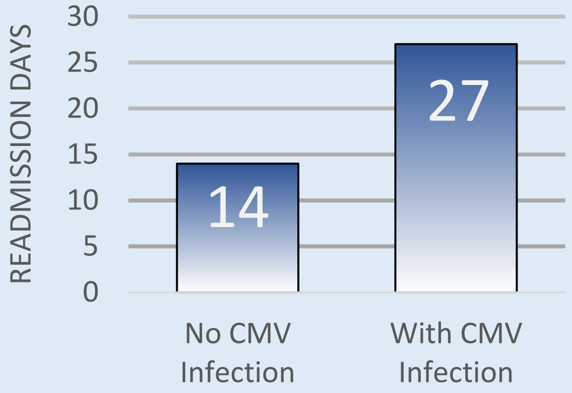

Conclusions: CMV infection was common, and associated with higher healthcare resource use
